# TP53 mutation in newly diagnosed acute myeloid leukemia and myelodysplastic syndrome

**DOI:** 10.1186/s13000-021-01162-8

**Published:** 2021-10-31

**Authors:** Pimjai Niparuck, Pornnapa Police, Phichchapha Noikongdee, Kanchana Siriputtanapong, Nittaya Limsuwanachot, Budsaba Rerkamnuaychoke, Suporn Chuncharunee, Teerapong Siriboonpiputtana

**Affiliations:** 1 Division of Hematology, Department of Medicine, Ramathibodi Hospital, Mahidol University, Bangkok, Thailand; 2 Human Genetics Laboratory, Department of Pathology, Ramathibodi Hospital, Mahidol University, Bangkok, Thailand

**Keywords:** TP53 mutation, Acute myeloid leukemia, Myelodysplastic syndrome, T-AML/MDS, complex karyotype, monosomy

## Abstract

**Objectives:**

TP53 mutation is found frequently in therapy related acute myeloid leukemia (AML)/ myelodysplastic syndrome (MDS), AML and MDS patients with monosomy or complex karyotype. However, the prevalence and treatment outcome in TP53 mutated AML/MDS patients in Asian population are scarce. We therefore conducted this study to analyze the prevalence and the treatment outcomes of TP53 mutation in AML and MDS-EB patients.

**Methods:**

Patients with newly diagnosed AML and MDS-EB were recruited, extraction of deoxyribonucleic acid from bone marrow samples were done and then performing TP53 mutation analysis, using MassArray® System (Agena Bioscience, CA, USA).

**Results:**

A total of 132 AML/MDS patients were recruited, patients with de novo AML, secondary AML, MDS-EB1, MDS-EB2 and T-AML/MDS were seen in 66, 13, 9, 9 and 3%, respectively. TP53 mutation was found in 14 patients (10.6%), and prevalence of TP53 mutation in T-AML/MDS, secondary AML, de novo AML and MDS-EB patients were 50, 17.6, 9.2 and 8%, respectively. Three patients had double heterozygous TP53 mutation. Mutated TP53 was significantly detected in patients with monosomy and complex chromosome. Common TP53 mutation were R290C, T220C, A249S and V31I which V31I mutation was reported only in Taiwanese patients. Most variant allele frequency (VAF) of TP53 mutation in the study were greater than 40%. Three year-overall survival (OS) in the whole population was 22%, 3y-OS in AML and MDS-EB patients were 22 and 27%, respectively. The 1y-OS in patients with TP53-mutant AML/MDS were shorter than that in TP53 wild-type patients, 14% versus 50%, *P* = 0.001. In multivariate analysis, factors affecting OS in 132 AML/MDS patients was mutant TP53 (*P* = 0.023, HR = 1.20–7.02), whereas, WBC count> 100,000/μL (*P* = 0.004, HR = 1.32–4.16) and complex karyotype (*P* = 0.038, HR = 1.07–9.78) were associated with shorter OS in AML patients.

**Discussion:**

In this study, the prevalence of TP53 mutation in de novo AML and MDS-EB patients were low but it had impact on survival. Patients with monosomy or complex karyotype had more frequent TP53 mutation.

## Introduction

TP53 is located on chromosome site 17p13, the p53 protein is an important role in impeding cell cycle progression and induction of apoptosis. The loss or mutation of TP53 promotes the development of cancer including acute myeloid leukemia (AML) and myelodysplastic syndrome (MDS). The prevalence of TP53 mutation in de Novo AML, secondary AML, MDS-EB and therapy related AML/MDS (T-AML/MDS) are 5–10%, 4%, 15–60% and 30–50%, respectively [1–8]. Whereas, the TP53 mutation has been found in 9.4, 55 and 77% of MDS patients, MDS patients with complex karyotype and MDS patients with monosomy, respectively [7, 8]. Many various types of TP53 mutation have been reported in AML/MDS patients, nevertheless, the common TP53 mutations are located in the codon 175, 220, 248 and 273 [9, 10]. The correlation between variant allele frequency (VAF) of TP53 mutation and treatment outcome was previously reported that mutated TP53 with VAF > 40% had median overall survival of 124 days and VAF < 20% was not associated with complex karyotype and treatment outcome such as complete remission (CR) rate, overall survival (OS) or event free survival (EFS) rate [11, 12]. Moreover, according to a recent study by Bernald et al. that illustrates the treatment outcome is similar between monoallelic TP53 mutation and TP53 wild-type MDS patients, which monoallelic and biallelic TP53 mutation are found in one-third and two-third of MDS patients, respectively [13]. Currently, there are few clinical data about TP53 mutation in patients with AML and MDS with excess blasts (MDS-EB) in Asian countries [2, 6]. Hence we conducted this retrospective study to analyze the prevalence and the treatment outcomes of TP53 mutation in AML and MDS-EB patients.

## Materials and methods

### Patients and samples

Bone marrow deoxyribonucleic acid (DNA) samples from 132 consecutive patients with newly diagnosed AML and MDS-EB during May 2014–April 2018 were used for performing TP53 mutation analysis, using MassArray® System (Agena Bioscience, CA, USA). All AML and MDS-EB patients recruited in this retrospective study had the routine study results of nucleophosmin 1 (NPM1), FMS like tyrosine kinase (FLT3) internal tandem duplication (ITD) and CCAAT/enhancer- binding protein alpha (CEBPA) analysis.

### Chemotherapy protocol

Younger AML patients (aged< 60 years) received induction chemotherapy with intravenous (i.v.) Ara-C 100 mg/m^2^/day for 7 consecutive days together with i.v. idarubicin 12 mg/m^2^/day for 3 consecutive days (7 + 3 regimen). Patient aged≥ 60 years or unfit patients were treated with 5 + 2 regimen; 5 days of i.v. Ara-C 100 mg/m^2^/day combined with 2 days of i.v. idarubicin 12 mg/m^2^/day. The bone marrow (BM) study was re-evaluated 28 days after induction therapy. Patients achieving CR received the first cycle of consolidation chemotherapy with the same regimen as induction therapy and then followed by 3 cycles of IDAC or HiDAC therapy for patients aged< 70 years.

AML patients aged≥ 70 years and MDS-EB patients were treated with azacitidine 100 mg/day subcutaneously for 7 consecutive days and repeated every 4 weeks. Disease response was re-evaluated after 4–6 cycles of azacitidine. CR was defined as BM blasts< 5% with absolute neutrophil count≥ 1000/μL, platelet count≥ 100,000/μL, absence of circulating blasts and absence of extramedullary disease.

### Targeted genomic analyses

MassArray® System (Agena Bioscience, CA, USA) was utilized to perform targeted genomic confirmation in this study. Once the target sequence(s) were input, the software will automatically generate the set of three primers; forward, reverse & extension primer. Primers were designed to target hotspots mutation at codon 31, 126, 143, 175, 179, 194, 220, 238, 241, 248, 249, 255, 273, 280, 282, 290 and 310.

Multiplex PCR technique consisted of 3 steps;

- PCR amplification was done using polymerase chain reaction (PCR) primer mix (forward and reverse primers) at the concentration of 100 nM, MgCl2 solution, dNTP 500 μM, PCR buffer 1x concentration, PCR enzyme 0.2 unit/μL and 10–20 ng DNA. All to mix up to the total volume of 5 μL. The thermal cycles were 2 min-cycle of 95 °C following 30 s-cycle of 95 °C, 30 s-cycle of 56 °C, then 1 min-cycle of 72 °C = 45 cycles and 5 min-cycle of 72 °C = 5 cycles.

- Eliminating excess dNTPs from the previous step by adding shrimp alkaline phosphatase (SAP). The protocol comprised 10X SAP buffer 0.17 μL, SAP Enzyme 0.3 μL, distilled water (HPLC grade) 1.53 μL, to incubate in the thermal cycles of 37 °C = 40 min following by 85 °C = 5 min.

- Adding a single nucleotide as the terminator bases (ddNTPs) at the 3′ position of the extend primers. By bringing the PCR products from former step, to add with extend primer mix 0.52–1.57 μM, IPLEX® buffer 0.222x, IPLEX® terminator mix 0.222x and IPLEX® enzyme 0.142 unit/μL, to make up to the total volume of 9 μL. The thermal cycles are; [95 °C = 30 s + {95 °C = 5 s + 5x(52 °C = 5 s + 80 °C = 5 s)}] = 40 cycles following 72 °C = 3 min.

Since very small volume dispensed, either Automated Liquid Handler or Manual Dispensing was adopted by the Manufacturer’s certified technician.

- Data analysis report; to dispense the final analyze on the SpectroChip® and bring in to the MassArray® analyzer (Mass Spectrometry), the report generated via MassArray® Typer Software.

### Outcome assessment

The objectives of this study were to evaluate the prevalence of TP53 mutation in AML and MDS-EB patients, and also overall survival (OS). OS was defined as the interval between the dates of diagnosis and death.

### Statistical analysis

The factors which included age, white blood cell (WBC) count, cytogenetics, TP53 and other molecular data were compared between patients with and without TP53 mutation, using Fisher’s exact test. OS was calculated by the Kaplan–Meier method, difference between groups were calculated using the log-rank test for univariate analysis. Cox’s Regression model was used for multivariate survival analysis. All calculations were performed using the statistical package of social sciences software, SPSS statistics version 17 (Chicago: SPSS Inc.; 2008).

## Results

### Cytogenetic and molecular analysis

A total of 132 patients with newly diagnosed AML/MDS-EB were recruited into the study, and 57% of whom were male. A median age was 59 years, 48% of patients were older than 60 years. Patients with de novo AML (66%), secondary AML (13%), MDS-EB1 (9%), MDS-EB2 (9%) and T-AML/MDS (3%) were recruited into the study. Abnormal karyotype was seen in 51% of all AML/MDS patients that included complex karyotype (18%), monosomy (16%), other cytogenetic abnormalities (17%), abnormal chromosome 3 (3%) and isolated monosomy, t(8;21), trisomy 8 (with a frequency of 4.5% each). NPM1 and FLT3-ITD mutations were observed in 24 and 12% of 107 AML patients, respectively. Whereas only 3 out of 25 MDS patients had mutated NPM1.

(NPM1^mu^), FLT3-ITD (FL3-ITD^mu^) or CEBPA (CEBPA^mu^).

In the whole study, TP53 mutation was detected in 14 patients (10.6%), and it was often found in T-AML/MDS (50%) and secondary AML patients (17.6%). TP53 mutation was less frequently seen in MDS-EB including one patient with T-MDS-EB (8%), and the rate of TP53 mutation in nineteen high risk MDS patients (R-IPSS> 3.5) was 11%. Whereas the prevalence of mutant TP53 in de novo AML patients was 9.2%.

Patients’ characteristics are shown in Table 1. Median age of TP53 mutated AML/MDS patients was 66 years. In group of patients with TP53 mutation, 12 patients had abnormal karyotype which 9 of those patients had unfavorable cytogenetic risk. TP53 mutation was significantly seen in AML/MDS patients with abnormal karyotype (86%), especially monosomy (33%) and complex chromosome (29%). In addition, half of AML/MDS patients with TP53 mutation had complex chromosome and 71% of whom had complex karyotype with monosomy (Table 2).

**Table 1:** Patients’ characteristics and TP53 mutation in 132 patients with AML/ MDS

Factors	Number of AML/ MDS patients N (%)	TP53 mutated patients N (%)	*P*
Age, range 16–93 years (*N* = 132)			0.575
< 60 years	69 (52.3)	6/69 (8.7)	
> 60 years	63 (47.7)	8//63 (12.7)	
Disease (*N* = 132)			0.003
De novo AML	87 (65.9)	8/87 (9.2)	
Secondary AML	17 (12.9)	3/17 (17.6)	
MDS (excess blast)	24 (18.2)	1/24 (4.2)	
T-AML/MDS	4 (3.0)	2/4 (50)	
White blood cell count in AML patients (*N* = 107)			0.563
< 100,000/μL	88 (82.2)	12/88 (13.6)	
> 100,000/μL	19 (17.8)	0/19 (0)	
Chromosome analysis (*N* = 132)			0.016
Abnormal	67 (50.8)	12//67 (17.9)	
Normal	65 (49.2)	2/65 (3.1)	
Cytogenetic risk (*N* = 132)			0.01
Favorable	6 (4.5)	0/6 (0)	
Intermediate	89 (67.4)	5/89 (5.6)	
Unfavorable	37 (28)	9/37 (24.3)	
Complex chromosome (*N* = 132)			0.004
Yes	24 (18.2)	7/24 (29.2)	
No	108 (81.8)	7/108 (6.5)	
Complex with monosomy karyotype (*N* = 132)			0.008
Yes	14 (10.6)	5/14 (35.7)	
No	118 (89.4)	9/118 (7.6)	
Complex with del5/−5 or del7/−7 (*N* = 132)			0.026
Yes	12 (9.0)	4/12 (33.3)	
No	120 (91.0)	10/120 (8.3)	
Monosomy (*N* = 132)			0.002
Yes	21 (15.9)	7/21 (33.3)	
No	111 (84.1)	7/111 (6.3)	
Gene mutation (*N* = 107 AML patients)			0.893
FLT3-ITD	13 (12.1)	3/13 (23)	
NPM1	26 (24.3)	3/26 (11.5)	
CEBPA	9 (8.4)	0/9 (0)	
No mutation	80 (74.8)	10/80 (12.5)

**Table 2:** Types of TP53 mutation and treatment outcome in 14 AML/ MDS patients

No	Gender	Age (y)	Disease	Chromosome/ gene mutations	TP53 mutation type (% variant allele frequency)	Treatment regimen (status)	Time from diagnosis to death (Mo)
1	F	57	T-AML	46,XX,del(5)(q15q33),inv.(7)(p13p22),der(11) NPM1^wt^/FLT3^wt^/CEBPA^wt^	A249S (17.7)	untreated (dead)	0.5
2	F	68	T-MDS (EB)	44 ~ 46,XX,add(5)(q11.2),-7,del(7)(q22), dic(7;14)(q11.2;p13),add(12)(q24.3),-18 NPM1^wt^/FLT3^wt^/CEBPA^wt^	T220C (41.3), R290C (58.7)	azacitidine (dead)	4
3	F	64	T-AML	46,XX,t(1;10)(p13;p13),-5,add(5)(q31),add12(p13) NPM1^wt^/FLT3^wt^/CEBPA^wt^	R248W (44.3)	untreated (dead)	3
4	M	32	AML	46,XY,del(12)(p11),i(8)(q10),der(10),del(1)(p21) NPM1^wt^/FLT3^wt^/CEBPA^wt^	V31I (87.3) S241F (19.4)	7/3 (dead)	5
5	F	59	AML	46,XX,add19(p13.3) NPM1^wt^/FLT3^wt^/CEBPA^wt^	R290C (41.6)	7/3 (dead)	4
6	M	71	AML	45,XY,-10,-12,-20,+ 8,+ 9 NPM1^wt^/FLT3^wt^/CEBPA^wt^	T220C (48)	untreated (dead)	1
7	M	43	AML	42,XY,der(1)del(1)(p13p22),-3,der(5)t(5;15) (q11.2;q11.2),-7,add(8)(q24.1),-12,-15,add(8),der(8) NPM1^wt^/FLT3^wt^/CEBPA^wt^	C238Y (43)	7/3 (dead)	12
8	M	74	MDS-EB	45,XY,-7 NPM1^wt^/FLT3^wt^/CEBPA^wt^	A249S (14.2)	azacitidine (alive)	19
9	M	77	Secondary AML	44,XY,del(3)(q21q27),-3,-12,-18,+ 21 **NPM1**^**mu**^**/FLT3-ITD**^**mu**^**/ CEBPA**^**wt**^	R282W (100)	untreated (dead)	1
10	F	42	AML	45,XX,-7 NPM1^wt^/FLT3^wt^/CEBPA^wt^	T220C (52.7) R290C (35.8)	untreated (dead)	1
11	M	42	AML	47,XY,+ 1 **NPM1**^**mu**^**/FLT3-ITD**^**mu**^**/ CEBPA**^**wt**^	V31I (47.7)	7/3 (dead)	6
12	M	68	Secondary AML	46,XY NPM1^wt^/FLT3^wt^/CEBPA^wt^	V31I (23.1)	untreated (dead)	1
13	M	88	AML	47,XY,+ 8 NPM1^wt^/FLT3^wt^/CEBPA^wt^	R290C (66.2)	untreated (dead)	4
14	M	71	Secondary AML	46,XY **NPM1**^**mu**^**/FLT3-ITD**^**mu**^**/ CEBPA**^**wt**^	A249S (11.3)	azacitidine (dead)	16

In AML patients, TP53 mutation was found in 6 out of 18 AML patients with complex karyotype and 4 out of 16 AML patients with monosomy. Co-occurring TP53 with NPM1 and FLT3-ITD mutations was detected in 2 secondary AML and 1 de novo AML patients. None of AML/MDS patients with favorable cytogenetic risk or CEBPA mutation had mutated TP53. The median WBC in twelve AML and T-AML patients with mutant TP53 were 19,200/μL (range, 2670- 83,000/μL). The common TP53 mutations were R290C (29%) and T220C, A249S, V31I (with a frequency of 21% each). The other codons that we found TP53 mutation were 248, 241, 238 and 282. Three patients had double heterozygous TP53 mutation. Except for mutated TP53 in codon 249 and 241, the variant allele frequency (VAF) of TP53 mutation in the remaining codons were greater than 20%. Patients’ characteristics and the types of TP53 mutation are shown in Table 2.

### Treatment outcomes

**Fig. 1:**
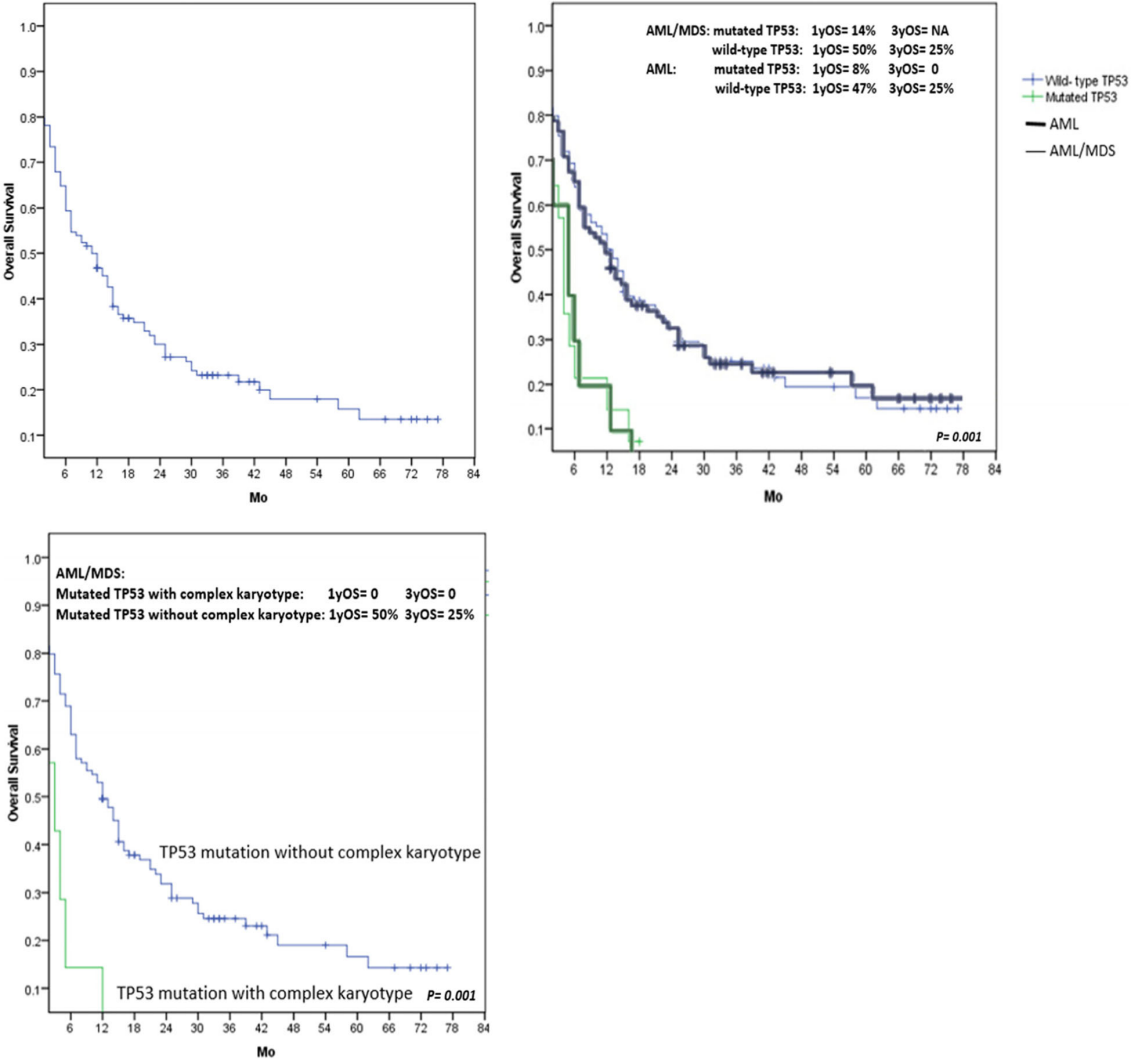
**A**) OS in patients with AML/MDS **B**) OS in AML/MDS and AML patients with and without TP53 mutation **C**) OS in TP53mutated AML patients with and without complex karyotype

Of 132 patients, 102 patients received treatment included 7/3 (72 patients), 5/2 (13 patients) and azacitidine (17 patients). Sixty-one treated patients (60%) achieved CR and only 11 CR patients (18%) underwent allogeneic stem cell transplantation. 3y-OS in the whole population was 22%, 3y-OS in AML and MDS-EB patients were 22 and 27%, respectively. In the whole study population, the factors affecting OS in the univariate analysis were unfavorable chromosome risk, complex karyotype, monosomy, mutant TP53 and mutant TP53 with complex chromosome or monosomy, nevertheless, only mutant TP53 (*P* = 0.023, HR = 1.20–7.02) was significantly associated with shorter OS in the multivariate analysis. The survival of patients with mutated TP53 and wild-type TP53 are shown in Fig. 1. Whereas, WBC count> 100,000/μL, unfavorable cytogenetic risk, complex karyotype, monosomy and TP53 mutation were significantly associated with shorter OS in patients with AML according to the univariate analysis but the multivariate analysis indicated that poorer OS was found only in AML patients with WBC count> 100,000/μL (*P* = 0.004, HR = 1.32–4.16) and complex karyotype (*P* = 0.038, HR = 1.07–9.78). Parameters related to the survival of AML/MDS patients are shown in Table 3.

**Table 3:** Parameters affecting overall survival in 132 patients with AML/MDS

AML/ MDS patients				AML patients			
Factors	1y- OS	3y- OS	*P*	Factors	1y- OS	3y- OS	*P*
AML	44	22	0.535	WBC < 100,00/μL	46	27	0.014
MDS	60	27		WBC > 100,00/μL	28	0	
Complex karyotype			< 0.001	Complex karyotype			< 0.001
Yes	17	0		Yes	6	0	
No	54	29		No	51	27	
Monosomy			0.03	Monosomy			0.003
Yes	19	7		Yes	13	6	
No	53	27		No	49	25	
Complex karyotype with monosomy			< 0.001	Complex karyotype with monosomy			0.001
Yes	14	0		Yes	10	0	
No	51	27		No	47	25	
Chromosome			0.004	Chromosome			0.023
Favorable	80	40		Favorable	75	38	
Intermediate	54	28		Intermediate	50	27	
Unfavorable	27	8		Unfavorable	22	11	
TP53			0.001	TP53			0.001
Mutant TP53	14	NA		Mutant TP53	8	0	
Wild- type TP53	50	25		Wild- type TP53	47	25	
Mutated TP53 with complex karyotype			0.001	Mutated TP53 with complex karyotype			0.004
Yes	0	0		Yes	0	0	
No	50	25		No	46	23	
Mutated TP53 with monosomy			0.138	Mutated TP53 with monosomy			0.034
Yes	17	NA		Yes	0	0	
No	48	24		No	45	23	

*Abbreviation*: *NA* not available

## Discussion

Except for secondary AML and MDS-EB groups, the prevalence of TP53 mutation in this study was similar to the previously published data [1–6]. We found high prevalence of TP53 mutation in patients with secondary AML (17%) and low prevalence of TP53 mutation in MDS-EB patients. In this study, the prevalence of TP53 mutation in the whole MDS-EB group was lower than that seen in the previous studies, 8% versus 15–60% [7, 8]. Nevertheless, the rate of TP53 mutation increased to 14% in MDS-EB patients with complex karyotype. The prevalence of TP53 mutation in 19 patients with high-risk MDS was 11% compared with 15% (4 out of 26 patients) in the previous study [14]. The possible causes of low prevalence of TP53 mutation in our MDS-EB patients might be from the small number of MDS-EB patients in the study (25 patients), low incidence of complex karyotype (7 patients) and/or underestimate the true mutation prevalence since our PCR technique was designed to detect only multiple hotspot mutations that was not the whole gene. However, the prevalence of TP53 mutation in Asian MDS and AML patients were 10.2–13% and 5.4–7%, respectively [2, 15–19]. Whereas, TP53 mutation was found in 9% of Chinese patients with MDS-EB [16]. TP53 mutation was frequently found in complex karyotype patients (42–59%) and was associated with poor overall survival in Asian patients with MDS and AML. [2, 15–19]. The prevalence, cytogenetic abnormality and prognosis of AML or MDS patients with mutated TP53 in our study were similar to the data in Asian AML/MDS patients. All TP53 mutated AML patients in the study had WBC count less than 100,000/μL and mutant TP53 was not detected in AML patients with favorable cytogenetic risk. Five out of seven de novo AML patients with TP53 mutation were younger than 60 years (range, 32–59 years), whereas, all secondary AML patients were older than 60 years. Only few patients had co-occurrence Mutations of TP53 with NPM1 and FLT3-ITD. Although the DNA probe array was designed to detect TP53 mutation covering 17 codons, we found mutation occurred in only 8 codons (31, 220, 238, 241, 248, 249, 282 and 290). We also demonstrated the uncommon patterns of TP53 mutation which occurred in codon 31 (V31I) and 249 (A249S), these two mutations have been illustrated in only few previously studies [2, 20]. However, there was no report of these mutations in large cohort studies of AML/MDS patients in Western countries [4, 6, 11, 13, 21, 22]. The most common type of TP53 mutation in this study was R290C (28.6%), but interestingly, we found high prevalence of A249S and V31I mutation, which V31I mutation was observed with higher rate than the other types of TP53 mutation in Taiwanese patients with AML [2]. TP53 mutation in codon 175 and 273 were not found in our AML/MDS patients, and only 1 patient had TP53 mutation in codon 248, these 3 mutation types were commonly seen in AML patients in the previous studies [9, 10, 13]. The another common codon of TP53 Mutation (codon 220) was also detected in our study (21.4%) [13]. In this study, A249S was observed in patients with MDS-EB, secondary AML from MDS or T-AML but was not found in de novo AML patients, and it occurred in patients with either normal or abnormal karyotype. Whereas V31I was seen in either de novo or secondary AML, and was not associated with cytogenetic patterns. Except for TP53 mutation in codon 249 and 241, most VAF of TP53 mutation in our patients were greater than 40%. The survival was significantly shorter in TP53 mutated AML/MDS patients compared with those in TP53 wild-type patients. Nevertheless, the actual survival time for patients with TP53 mutation should be longer than our TP53 mutated patients’ survival since half of these patients in the study had serious infection at the time of diagnosis and didn’t receive any treatment. The other limitations of this study were small number of both AML/MDS patients and TP53 mutated patients and the PCR technique was designed to detect only hotspot mutations, even seventeen mutational hotspots were sequenced. Study of whole TP53 gene mutation might increase the prevalence of TP53 mutation in our patients slightly from detection of other uncommon TP53 mutations. Finally, the MassArray technique would be useful for routine screening TP53 mutation in AML/MDS patients in the medical center that the next generation sequencing is not available and the cost of this testing is not high.

## Conclusion

The prevalence of TP53 mutation in de novo AML and MDS-EB patients were low but it had impact on survival. Patients with monosomy or complex karyotype had more frequent TP53 mutation.

## Data Availability

Not applicable.

## Abbreviations

MDS-EB: MDS with excess blasts
T-AML: Therapy related AML
T-MDS: Therapy related MDS
FLT3-ITD: FMS related tyrosine kinase 3-internal tandem duplications
NPM1: Nucleophosmin1
CEBPA: CCAAT/enhancer binding protein α gene
